# Autism Link to Herpes Simplex Virus 2 Antibody in Pregnancy Likely To Be Spurious

**DOI:** 10.1128/mSphere.00106-17

**Published:** 2017-03-29

**Authors:** Amalia S. Magaret, Anna Wald

**Affiliations:** a Departments of Laboratory Medicine and Biostatistics, University of Washington, Seattle, Washington, USA; b Departments of Medicine, Epidemiology and Laboratory Medicine, University of Washington, Seattle, Washington, USA; University of Michigan

**Keywords:** autism, herpesviruses

## LETTER

We are writing to express our concerns regarding the publication of the article entitled "Maternal Immunoreactivity to Herpes Simplex Virus 2 and Risk of Autism Spectrum Disorder in Male Offspring" (1).

First, a correlation between new herpes simplex virus (HSV) acquisition, or HSV reactivation, and antibody levels has not been established, and thus high antibody levels should not be taken as a proxy for viral replication or infection severity. Second, the immunoassay used in this study is intended for qualitative diagnosis of *Toxoplasma gondii*, *Rubella*, cytomegalovirus, and HSV-1 and -2 (ToRCH) infections and not for comparison of quantitative antibody levels (2). To the point, the Zeus HSV package insert states “The numeric value of the result above the cutoff is not indicative of the amount of anti-HSV-2 IgG antibody present.”

However, the statistical analysis generated our greatest concern, given the number of tests performed on this data set. An adjustment for multiple comparisons was performed once for the 5 pathogens but did not address the number of statistical tests applied uniquely to HSV-2. We counted 12 separate tests of association between HSV-2 antibody level and eventual development of autism spectrum disorder (ASD) in boys alone. Mahic et al. examined evidence of HSV-2 antibody in maternal plasma and found that women who had evidence of herpes simplex virus infection either at midpregnancy or at delivery were no more likely to have children with ASD (2 statistical tests [one at each time point]). They continued searching and found that median levels of antibody to HSV-2 in women also had no association with ASD in boys after multiplicity adjustment either at midpregnancy or at delivery (2 statistical tests). In a third try, they reported four more potential thresholds of antibody level and emphasized one of the four, above which a few mothers of boys had an elevated risk of ASD (8 more tests [four at each time point]). The sheer number of tries that it took to come up with any association between HSV-2 and ASD makes their finding extremely unlikely to be reproduced.

Nearly 15% of women of childbearing ages are estimated to be HSV-2 seropositive globally (3). Thus, an astounding number of women globally will experience a pregnancy while HSV-2 seropositive. The article by Mahic et al. has a potentially dramatic impact on the well-being of many women. The message of this paper should be tempered to reflect the poor evidence which it comprises.
